# MSF-CPMP: a novel multi-source feature fusion model for prediction of cyclic peptide membrane permeability

**DOI:** 10.1093/bib/bbaf631.072

**Published:** 2025-12-12

**Authors:** Zimeng Chen, Yijun Zhang, Zhuxuan Wan, Qianhui Jiang, Xiaoling Lu, Bin Yan, Jing Qin, Yong Liu, Junwen Wang

**Affiliations:** Division of Applied Oral Sciences & Community Dental Care, Faculty of Dentistry, The University of Hong Kong, 34 Hospital Road, Hong Kong SAR, China; Guangxi University for Nationalities, Nanning, China; Guangxi University for Nationalities, Nanning, China; Division of Applied Oral Sciences & Community Dental Care, Faculty of Dentistry, The University of Hong Kong, 34 Hospital Road, Hong Kong SAR, China; Division of Applied Oral Sciences & Community Dental Care, Faculty of Dentistry, The University of Hong Kong, 34 Hospital Road, Hong Kong SAR, China; Division of Applied Oral Sciences & Community Dental Care, Faculty of Dentistry, The University of Hong Kong, 34 Hospital Road, Hong Kong SAR, China; School of Pharmaceutical Sciences (Shenzhen), Shenzhen Campus of Sun Yat-sen University, Shenzhen, Guangdong, China; Guangxi University for Nationalities, Nanning, China; Division of Applied Oral Sciences & Community Dental Care, Faculty of Dentistry, The University of Hong Kong, 34 Hospital Road, Hong Kong SAR, China

## Abstract

**Motivation:**

Membrane permeability represents a critical bottleneck in cyclic peptide drug development, limiting the clinical translation of these therapeutically attractive molecules despite their inherent stability and structural diversity. Current computational models for predicting cyclic peptide membrane permeability (CPMP) exhibit insufficient accuracy for early-stage drug screening, hampering the efficiency of lead optimization in pharmaceutical pipelines.

**Methodology:**

In this study, we introduce a novel multi-source feature fusion model called MSF-CPMP, which aims to increase the accuracy of predicted CPMP. The MSF-CPMP model incorporates three features extracted from SMILES sequences, graph-based molecular structures, and physicochemical properties of cyclic peptides.

**Results:**

By benchmarking with other machine learning and deep learning-based methods, MSF-CPMP achieved the highest levels of the evaluation metrics such as accuracy of 0.9062 and AUROC of 0.9546, and further validated MSF-CPMP robustness in learning capabilities and efficacy of its multi-source fusion. MSF-CPMP has been validated on FDA-approved cyclic peptide therapeutics, demonstrating strong predictive power for clinical candidates. Our result demonstrates that MSF-CPMP outperforms other methods in predicting CPMP, providing a practical computational tool for accelerating drug screening and reducing attrition rates in cyclic peptide drug discovery, thereby advancing precision-guided pharmaceutical development.

**Availability:**

Code is available at https://github.com/wanglabhku/MSF-CPMP

